# Mesalazine-related lung disease in a patient with ulcerative colitis: A case report

**DOI:** 10.1097/MD.0000000000013242

**Published:** 2018-11-30

**Authors:** Po-Han Huang, Chia-Jung Kuo, Chang-Wei Lin, Yu-Ming Cheng, Han-Chung Hu, Chun-Yen Lin, Ming-Yao Su, Cheng-Tang Chiu

**Affiliations:** a Department of Gastroenterology and Hepatology, Chang Gung Memorial Hospital; b Chang Gung University, College of Medicine; c Department of Pulmonary and Critical Care Medicine; d Department of Internal Medicine, Chang Gung Memorial Hospital, Taoyuan, Taiwan.

**Keywords:** lung manifestation, mesalazine, ulcerative colitis

## Abstract

**Rationale:**

Mesalazine is widely used to treat inflammatory bowel disease (IBD). However, discriminating between pulmonary manifestations of IBD and drug-related lung disease remains a challenge. There were few case reports of mesalazine-related organizing pneumonia so far.

**Patient concerns:**

A 75-year-old female was diagnosed with ulcerative colitis and took mesalazine over a period of 2 years and 8 months. She presented with progressive shortness of breath for 3 days and visited our emergency department. Chest radiography showed increased bilateral infiltrates. During hospitalization her clinical condition deteriorated, and she was transferred to our intensive care unit under noninvasive ventilator support.

**Diagnosis:**

Computed tomography (CT) scan showed diffuse peribronchial and subpleural consolidations in bilateral lungs. Possible etiologies of interstitial lung disease were surveyed, including various infectious diseases and connective tissue diseases. Transbronchial lung biopsy showed characteristic features of organizing pneumonia.

**Interventions:**

Under the consideration of mesalazine-related lung disease, mesalazine was discontinued early in disease course and steroid therapy was given.

**Outcomes:**

The patient was discharged from hospital with improved clinical symptoms and radiographic images.

**Lessons:**

Although this patient suffered a life-threatening adverse event, prompt diagnosis with proper management can result in a favorable outcome.

## 1 Introduction

The prevalence and incidence of inflammatory bowel disease (IBD) vary geographically, with the highest rates reported in Western countries. However, the number of cases of IBD in Asia is increasing.^[1]^ Extraintestinal manifestations of IBD are common and require specific treatment depending on the affected organ, which are most frequently the skin, joints, and eyes; however, they can also less frequently involve other organs such as the liver, lungs, and pancreas.^[2,3]^

Mesalazine is a 5-aminosalicylic acid derivative and is widely used to treat IBD. Although mesalazine is generally well-tolerated, severe toxicity has been reported. Mesalazine-related interstitial lung disease included eosinophilic pneumonia, organizing pneumonia (OP), and nonspecific interstitial pneumonia.^[4–6]^ The onset of pulmonary disease from the introduction of mesalazine varied from days to several years.^[6]^ Discriminating between pulmonary manifestations of IBD and drug-related lung disease remains a challenge.

Mesalazine-related OP had been rarely reported.^[5,7]^ We present a case of a 75-year-old female with ulcerative colitis who developed progressive dyspnea and subsequent respiratory failure owing to mesalazine-related OP.

## 2 Case report

A 75-year-old nonsmoking woman presented to our emergency department due to progressive shortness of breath for 3 days. She denied fever, abdominal pain, diarrhea, bloody stool, or tenesmus. She had been diagnosed with ulcerative colitis at our hospital and received mesalazine 2 g per day for 2 years and 8 months.

Her initial vital signs were a blood pressure of 131/79mm Hg, pulse rate of 80 bpm, respiratory rate of 28/min and body temperature of 36.0°C. On physical examination, chest auscultation revealed coarse breathing sounds with bilateral crackles. Laboratory investigations revealed a white blood cell count of 10,600/μL with 79% neutrophils, hemoglobin level of 9.7 g/dL, and platelet count of 464,000/μL. Biochemistry profile showed an elevated level of C-reactive protein (79.7 mg/L), mildly impaired renal function (blood urea nitrogen level of 21.2 mg/dL and creatinine level of 1.23 mg/dL), and a normal alanine aminotransferase level (13 U/L).

**Figure 1:**
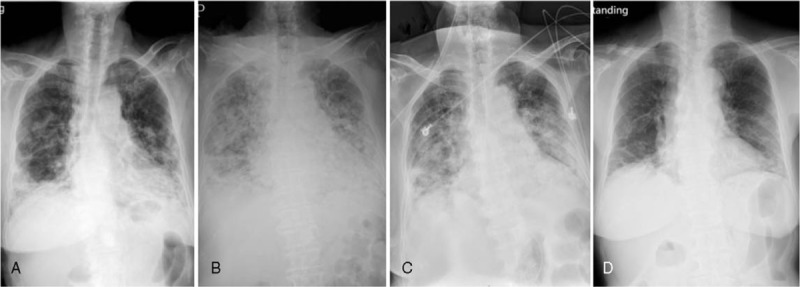
Serial chest X-rays showed: (A) infiltrates and consolidations in bilateral lungs with lower lobe predominance on the first day in the emergency department; (B) deterioration of bilateral lungs, for which noninvasive ventilator support was used; (C) improvement in bilateral infiltrates 1 week after discontinuing mesalazine and the initiation of steroid therapy; (D) resolution of most infiltrates in bilateral lungs about 2 months after the onset of symptoms.

Electrocardiography showed a normal sinus rhythm, and echocardiography of her heart was normal (left ventricular ejection fraction of 77%). Chest radiography revealed increased infiltration with patchy consolidations in both lungs and lower lobe predominance (Fig. 1A). She was then given oxygen via a nasal cannula. Under the impression of community-acquired pneumonia, she received antibiotic treatment and was admitted to our chest ward.

**Figure 2:**
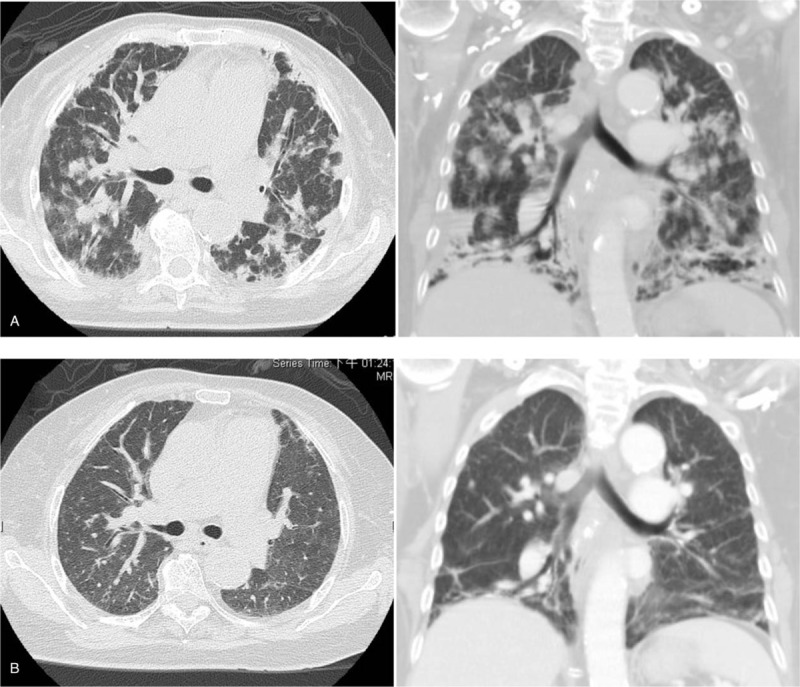
A computed tomography scan revealed: (A) diffuse peribronchial and subpleural consolidations in bilateral lungs with disease progression; (B) resolution of most infiltrates 3 months later.

Episodic high fever was noted since admission, and chest radiography showed progression of bilateral infiltrates. Further investigations were thus warranted, and a computed tomography (CT) scan showed diffuse peribronchial and subpleural consolidations in bilateral lungs with minimal interstitial thickening. The differential diagnosis included cryptogenic OP, acute interstitial pneumonia, and metastasis (Fig. 2A). In the following days, her respiratory condition deteriorated, and she was given noninvasive ventilator support (bi-level positive airway pressure, BiPAP) (Fig. 1B). Under the consideration of mesalazine-induced OP, mesalazine was discontinued on the 8th day of admission, and intravenous hydrocortisone 100 mg Q6H was started. She was then transferred to our medical intensive care unit.

We performed infection surveys for viruses, fungi, and mycobacteria. Due to positive cytomegalovirus (CMV) serologic tests including both IgM and IgG, intravenous ganciclovir was given for 7 days until the results of CMV antigenemia assays and qualitative CMV polymerase chain reaction (PCR) assays were negative. We also surveyed her autoimmune condition and vasculitis markers, and positive results of antinuclear antibodies and p-ANCA (antineutrophilic cytoplasmic antibodies, perinuclear pattern) were attributed to ulcerative colitis.

Her respiratory condition improved in the following days, although only a mild improvement was shown on serial chest plain films (Fig. 1C). Hydrocortisone 100 mg Q6H was maintained for 8 days and then tapered. BiPAP was shifted to an oxygen mask in the 2nd week of ICU admission.

**Figure 3:**
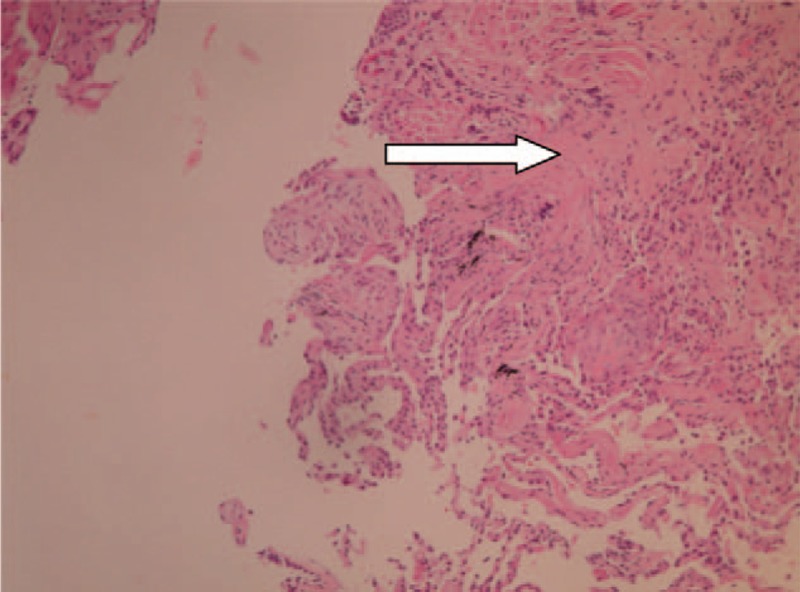
A transbronchial lung biopsy showed mild chronic inflammation with organizing pneumonia (arrow).

Bronchoscopy with bronchoalveolar lavage (BAL) was performed, and a specimen was sent for bacterial, fungal, and viral cultures, PCR testing of tuberculosis and *Pneumocystis jirovecii*, galactomannan, total and differential cell counts, CD4/CD8 ratio, and cytology. The results showed 81% macrophages, 10% lymphocytes, 8.2% neutrophils, and 0% eosinophils, with CD4 and CD8 counts of 24.6% and 22.7%, respectively (CD4/CD8 ratio: 1.08). The BAL culture showed growth of *Actinomyces odontolyticus*, which may have been caused by aspiration of oropharyngeal secretion. A transbronchial lung biopsy was done, and the pathology report showed mild chronic inflammation with OP (Fig. 3). There was no evidence of granuloma, malignancy, or vasculitis.

The patient was discharged on the 28th day of hospitalization with oral prednisolone 20 mg per day. She was regularly followed up at our chest out-patient department. One month after discharge, she had greatly reduced exertional dyspnea, and a chest plain film showed substantial improvements (Fig. 1D). A follow-up chest CT scan 3 months after disease onset showed resolution of most of the infiltration (Fig. 2B).

## 3 Discussion

Pulmonary disease associated with IBD has been observed for decades; however, it is still under-recognized. Discriminating between pulmonary manifestations of IBD and drug-related lung disease remains a challenge.

Pulmonary manifestations of IBD include airway disease, parenchymal disease, serositis, and pulmonary vascular disease. The large airways are the most common location of IBD involvement, and bronchiectasis is the most common pulmonary manifestation of IBD.^[8]^ The European Crohn and Colitis Organization Guidelines stated that pulmonary function tests are frequently abnormal in patients with IBD, and that latent interstitial lung disease may exist in 20% to 55% of patients with IBD.^[2]^ Drug-related lung disease and infection should always be an initial consideration when facing parenchymal disease of the lungs in patients with IBD.

The diagnosis of OP requires multidisciplinary approach, which includes careful history taking, medication review, radiographic images, and characteristic histopathologic features. CT scan typically demonstrates patchy consolidation or ground-glass opacity in subpleural or peribronchial areas.^[9]^ The characteristic histopathologic finding of OP shows intraluminal plugs of inflammatory debris occupying the more distal air spaces, and mild interstitial inflammation may be seen in surrounding lung.^[10]^ The diagnosis of mesalazine-related OP can be further made by excluding other secondary causes of OP, including other drugs or substance, autoimmune diseases, malignancies, infections, etc.^[11]^

By the literature review, there were only 6 case reports of mesalazine-related OP.^[5,7,12–15]^ While typical histopathologic finding is required preferably to establish the diagnosis of interstitial lung disease, only 4 of them had histologic diagnosis of OP.^[12–15]^ OP occurred in 3 to 6 months of mesalazine exposure in most cases; however, Shindoh et al reported a case of OP after 7.5-year mesalazine use, and the diagnosis was confirmed based on transbronchial lung biopsy and drug-induced lymphocyte stimulation test.^[13]^ Carratú et al reported a case of OP occurring 2 months following mesalazine use in a patient with Crohn disease (CD), but an extraintestinal manifestation of CD was favored due to concurrent relapse of CD and disease recovery despite maintenance of mesalazine.^[16]^

In our patient, the diagnosis of mesalazine-related OP was made based on characteristic findings from both radiographic images and histopathology as well as compatible clinical presentations, and we excluded possible etiologies including infections and connective tissue diseases. We did not favor pulmonary manifestations of IBD due to the lack of evidence of a relapse of ulcerative colitis such as diarrhea or bloody stool. We did not do a mesalazine rechallenge test due to the life-threatening adverse event; however, a drug rechallenge test may have been helpful to confirm the diagnosis. To establish the causal effect relationship of the drug-related adverse event, a Naranjo score of 5 was obtained, which suggested a probable causality.

This case report should serve to remind clinicians to be aware of possible adverse effects of medications and extraintestinal manifestations of IBD. Prompt diagnosis and proper management may lead to a favorable outcome.

## Author contributions

**Conceptualization:** Chia-Jung Kuo, Cheng-Tang Chiu.

**Data curation:** Chia-Jung Kuo, Chang-Wei Lin, Han-Chung Hu.

**Formal analysis:** Po-Han Huang, Chia-Jung Kuo, Chang-Wei Lin, Yu-Ming Cheng, Chun-Yen Lin, Ming-Yao Su, Cheng-Tang Chiu.

**Writing – original draft:** Po-Han Huang.

**Writing – review & editing:** Chia-Jung Kuo.
